# Supplementary material for: Short-term bioelectric stimulation of collective cell migration in tissues reprograms long-term supracellular dynamics
Source: PNAS Nexus. 2022 Mar 2;1(1):pgac002. doi: 10.1093/pnasnexus/pgac002 (PMC8962779; doi:10.1093/pnasnexus/pgac002)
Supplement: pgac002_Supplemental_Files [file pgac002_supplemental_files.zip › PNASNEXUS-PNASNEXUS-2021-00044-T-s06.pdf]

Supplementary Information for

Short-term bioelectric stimulation of collective cell migration in tissues reprograms long-term supracellular dynamics

Abraham E. Wolf[a], Matthew A. Heinrich[b,1], Isaac B. Breinyn[c,1], Tom J. Zajdel[b], and Daniel J. Cohen[b,2]

[a]Department of Chemical and Biological Engineering, Princeton University, Princeton, NJ 08544, USA;
[b]Department of Mechanical and Aerospace Engineering, Princeton University, Princeton, NJ 08544;
[c]Department of Quantitative and Computational Biology, Princeton University, Princeton, NJ 08544

[1]M.A.H. and I.B.B. contributed equally to this work contributed equally to this work.

[2]To whom correspondence should be addressed. Address: Attn. Daniel Cohen, 111 Hoyt Laboratory, Princeton, NJ 08544, USA. E-mail: danielcohen@princeton.edu

This PDF file includes:

Figures S1 to S9
Legends for Movies 1 to 6

Other supplementary materials for this manuscript include the following:

Movies 1 to 6

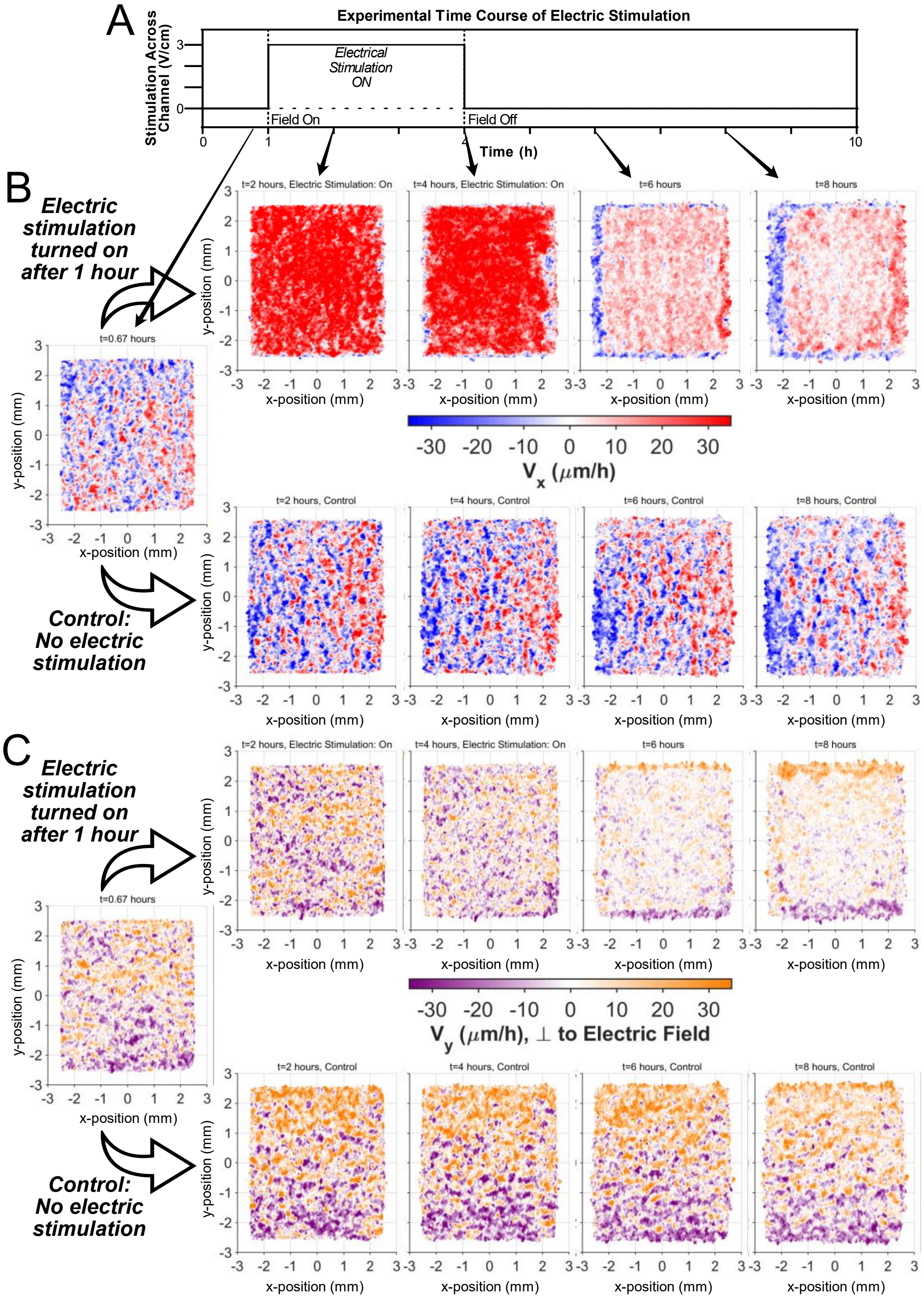

Fig. S1. V_x and V_y heatmaps for stimulated and control tissues. (A) Time course of our experimental setup, to see the complete step-response to bioelectric stimulation: 1 h of pre-stimulated control time; 3 h of stimulation ON; and 6 h of relaxation with stimulation OFF. **(B)** V_x heatmaps for stimulated and control tissues throughout the experiment. Stimulated tissue heatmaps showcase global directed motion during stimulation (strong red color), and retrograde motion in the edges after stimulation ends, with a pink center displaying ‘memory’ in the tissue bulk. **(C)** V_y heatmaps for stimulated and control tissues throughout the experiment. Stimulated tissue heatmaps have no global directed motion as seen in control tissues, however after stimulation ends, most of the motion is focused in the edge regions.

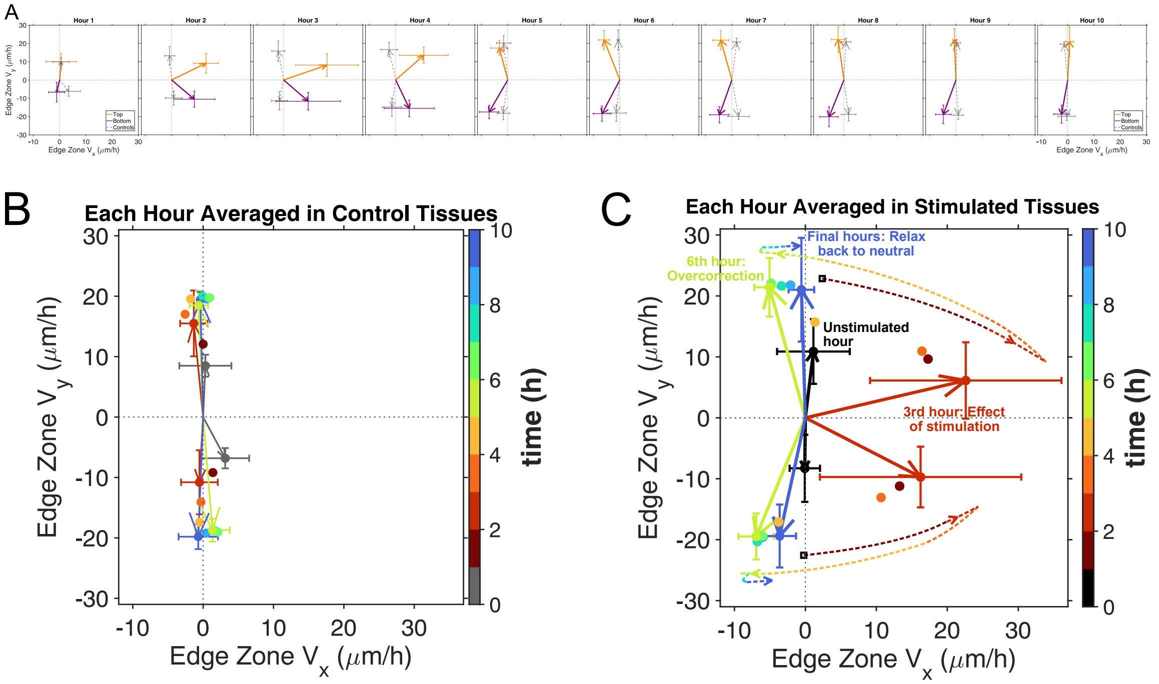

Fig. S2. Mean velocity vectors in the 150 μm-tall zones near the top and bottom edges show unique dynamics during and after stimulation. Stimulated tissue vectors exhibit similar behavior as the control before stimulation begins (first hour), obvious rightward motion during stimulation, then recoil opposite the direction of stimulation after it is turned off. **(A)** has 10 panels, each representing the average across the entire respective hour during the experimental time course. Electrical stimulation starts after the first panel, and ends after the fourth. In panels 2-4, the top (orange) and bottom (purple) average vector is strongly biased with the direction of stimulation. Immediately after, however, in panel 5, the vectors sharply flip back, overcorrecting toward the left. This overcorrection slowly relaxes over time through the 6 h post-stimulation period. Control vectors are provided in gray. **(B)** These same vectors are overlaid for the control tissue. Four vectors are shown, with the other six represented only by a filled-in circle. **(C)** Similar to panel B, but stimulated tissue data. Dashed lines and annotations were added to help visualize the development of the average vector over time, during the three phases of our experiment as described above. For all panels, all data was averaged over N=9 for stimulated tissues, N=6 for control tissues, and within a 150 μm-tall zone that excluded 1.5 mm on both the left and right for edge effects.

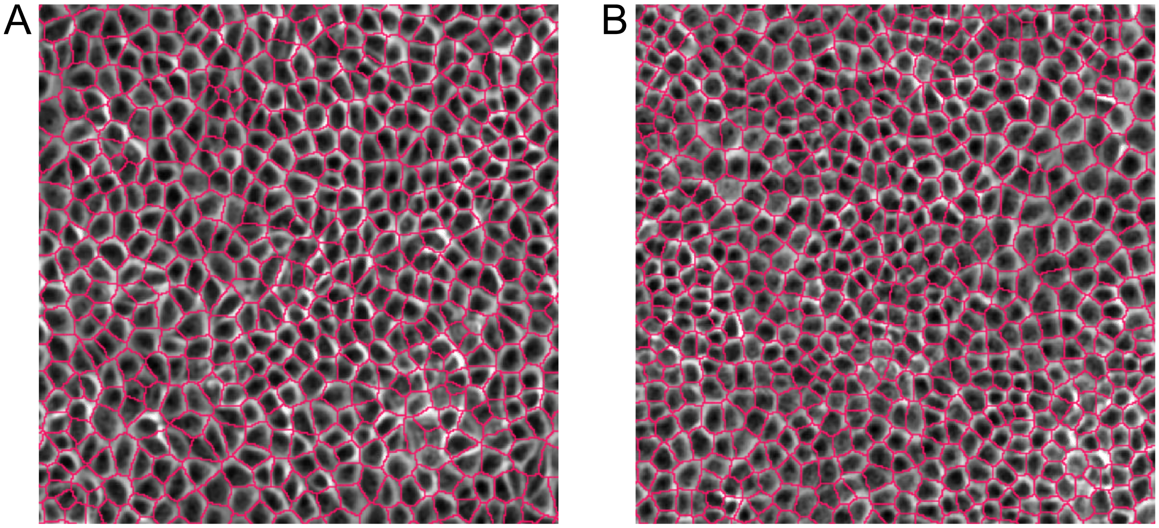

Fig. S3. Overlay of Voronoi tessellation produced using reconstructed nuclei and phase contrast microscopy of a 0.25 x 0.25 mm square area of an MDCK tissue, as described in Methods, showing sufficient accuracy both **(A)** during electrical stimulation and **(B)** several hours later after the tissue has become denser.

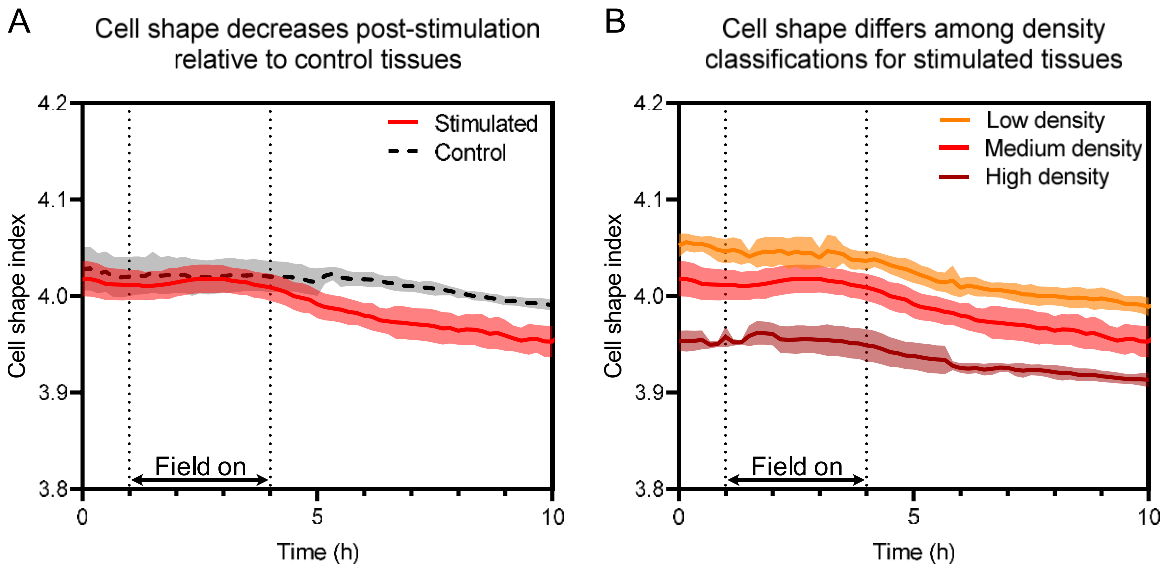

Fig. S4. Ensemble cell shape dynamics. Curves represent mean shape index (see Methods) of a 3 x 3 mm square in the center of MDCK tissues, averaged across all tissues. Shading represents standard deviation between replicate tissues. Each replicate tissue contained between 50,000 and 120,000 individual cells, but that number was not accounted for in the calculation of our reported error. Vertical dotted lines delineate electrical stimulation turning on ($t = 1$ h) and off ($t = 4$ h). **(A)** Medium density stimulated and control tissues exhibit similar cell shape dynamics before and during stimulation, however, mean cell shape decreases after stimulation ends for stimulated tissues (N=9 replicate tissues, each of which contained ~70,000 cells) relative to control (N=6). **(B)** Mean cell shape is inversely proportional to tissue density. All tissues show a stable mean cell shape before and during stimulation, but a steady decrease after stimulation ends. (Low density N=5 replicate tissues each with ~56,000 cells, medium density N=9 replicate tissues each with ~69,000 cells, high density N=4 replicate tissues each with ~111,000 cells.)

Cell counts show no signs of cell death due to stimulation

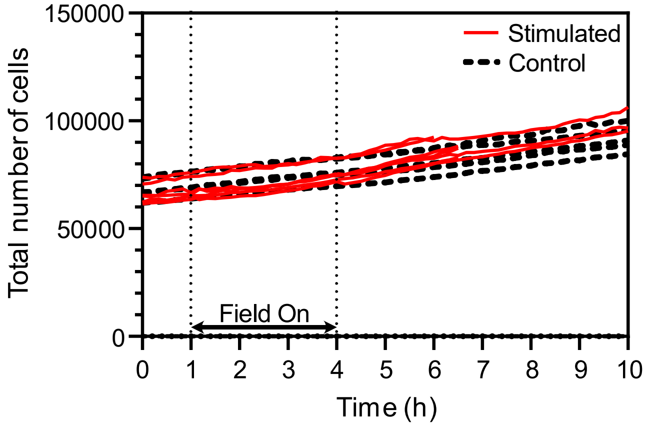

Fig. S5. Total cell counts for tissue replicates show no signs of cell death due to electrical stimulation. Curves show total cell counts across the entire tissue for both stimulated and control tissue replicates. Over time, cell division steadily increases the total population of cells in the tissue, with no apparent differences between control tissues and tissues that had undergone electrical stimulation.

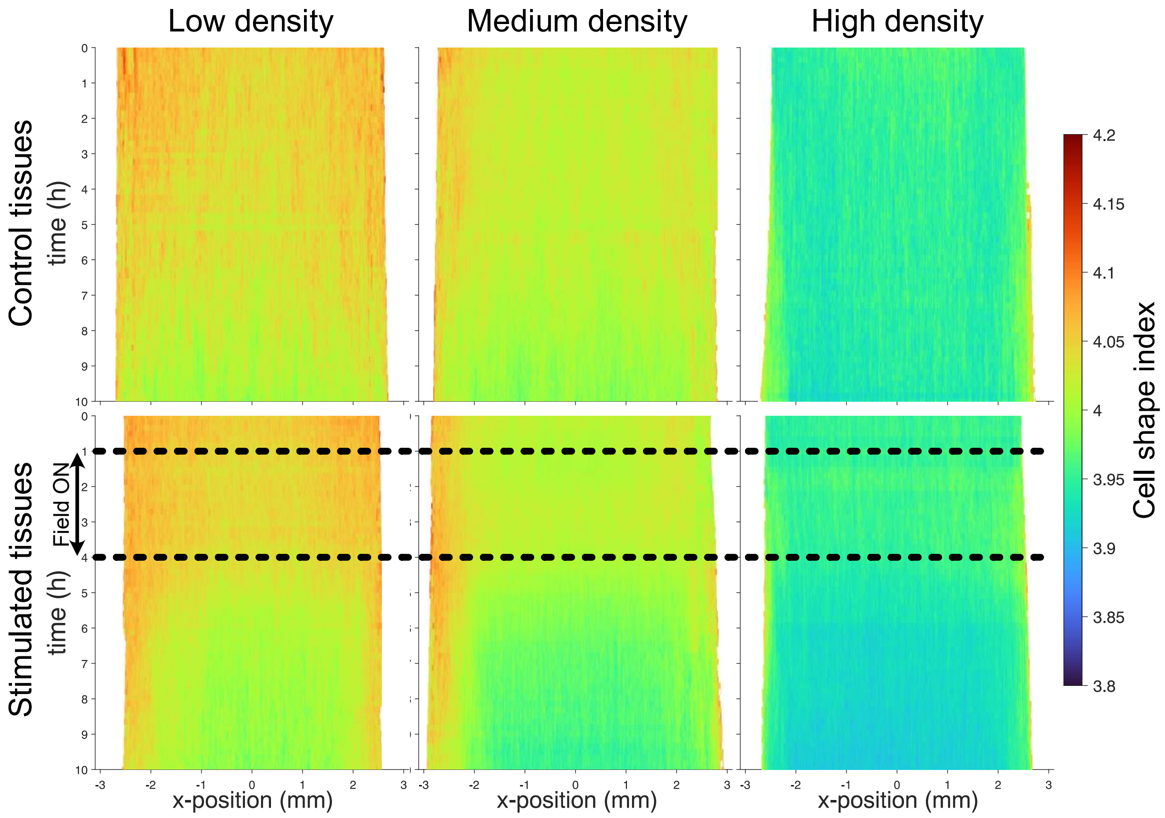

Fig. S6. Cell shape index kymographs show the spatial development of cell shape across the entire width of the tissue, for control and electrically stimulated tissues (shown by row), within the three categories of density analyzed—low, medium, and high (shown by column; see Methods). These data provide granularity of cell shape within the tissue during natural expansion and during our bioelectric assay, where the center of the tissue has a visible decline in cell shape index soon after electrical stimulation was turned off, even as the edges display similar patterns. Cell shape index was calculated for every cell (see Methods), numbering in the tens of thousands for each tissue, and then averaged vertically for the center 3 mm region of the tissue at every time point. Dashed dotted lines delineate electrical stimulation turning on (t = 1 h) and off (t = 4 h) in the stimulated tissues. (Low density: N=5 stimulated replicate tissues each with ~56,000 cells, medium density: N=9 stimulated replicate tissues each with ~69,000 cells, high density: N=4 stimulated replicate tissues each with ~111,000 cells. For control tissues, N=3, 6, and 2 respectively for low, medium, and high density control replicate tissues.)

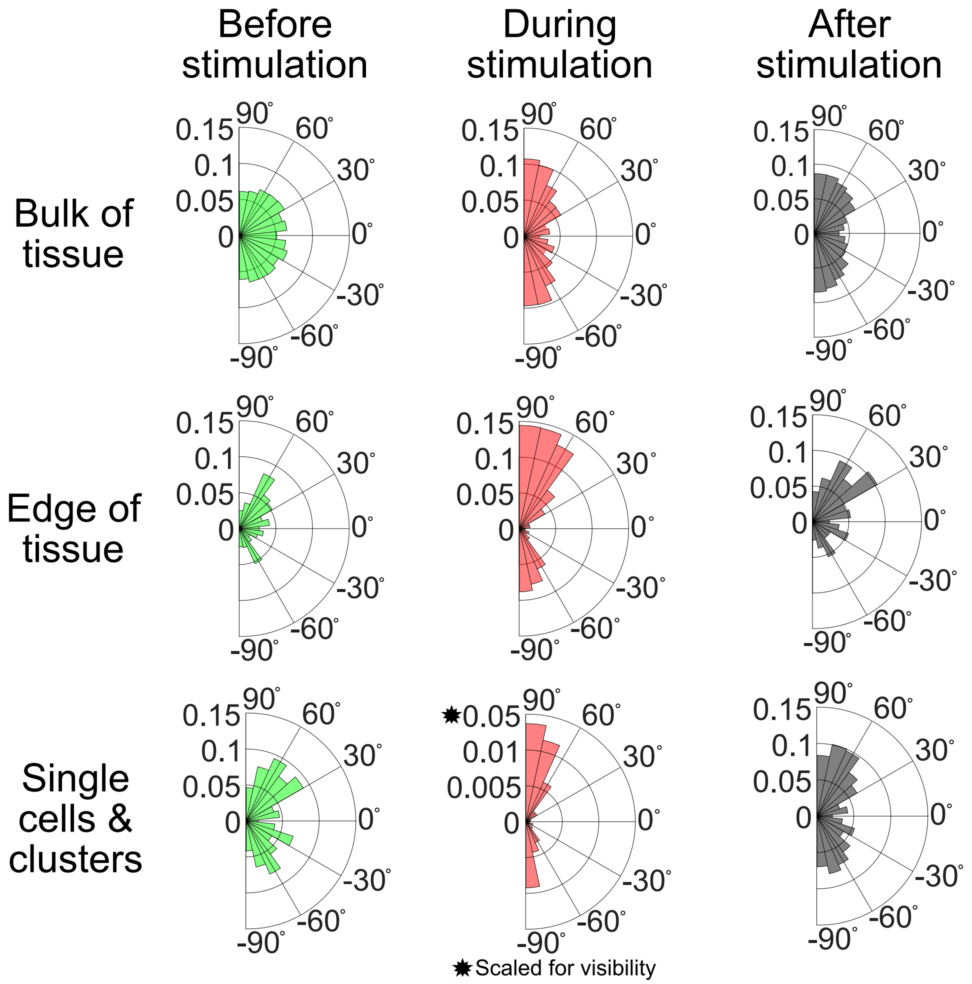

Fig. S7. Histograms of cell orientation (0º indicating rightward) for confluent tissue bulk (top row), confluent tissue edge (middle row), and single cells & clusters (bottom row), during our bioelectric assay—including before stimulation (green; t = 0 h), during stimulation (red; t = 3.5 h, after 2.5 h of stimulation), and long after stimulation (gray; t = 7.17 h, just over 3 h post-stimulation). All three cell regions show strong polarization perpendicular (vertical) to the field direction (horizontal) during stimulation, and a similar profile both before electrical stimulation and after the cells have relaxed post-stimulation. These data were calculated using FIJI's built-in 'OrientationJ' plug-in (see Methods) on the respective panels shown in Movie 5, time-lapse RFP fluorescence images of MDCK-II cells stably expressing E-cadherin:DsRed, which allowed cell orientation to be directly extracted from the unprocessed time-lapse images.

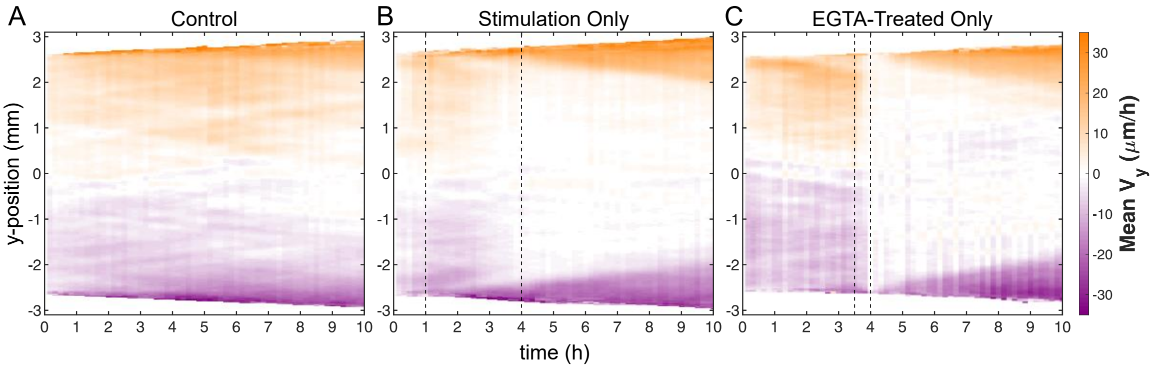

Fig. S8. Average kymographs across several tissues of mean V_y in **(A)** control (N=6), **(B)** stimulated (N=9), and **(C)** EGTA-treated (chemical junctional disruption, N=3) tissues. After their respective perturbations have concluded at t = 4 h, in both stimulated and EGTA-treated tissues, inward traveling waves of cell mobilization are clearly visible, just as demarcated in Figs. 4B-C for directionality order parameter kymographs. To calculate mean V_y across the entire height of the tissue, the vertical velocity vector components were averaged at each y location through the x-center of the tissue, ignoring 1.5 mm on the left and right for edge effects.

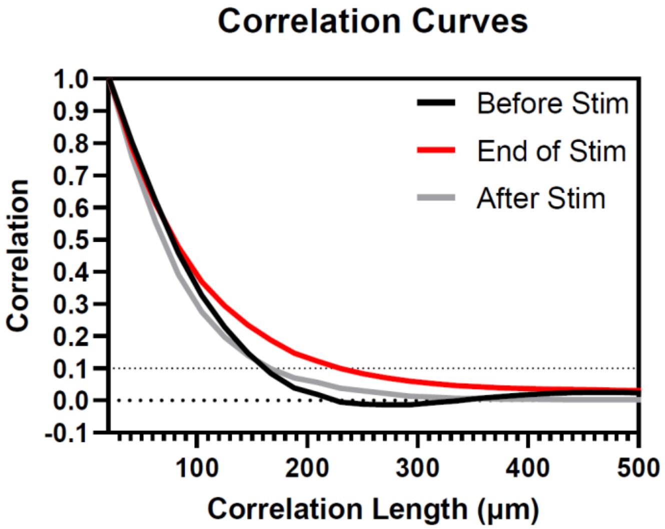

Fig. S9. Correlation curves at different periods in the experiment averaged over all stimulated tissues. The correlation curve at the end of stimulation (red) drops much slower than those before (black) and after (gray) stimulation, denoting a higher correlation length within the bulk of the tissue. (See Methods.)

Movie 1 (separate file). Phase-contrast time-lapse images of 5 x 5 mm MDCK tissues, side-by-side: a control tissue on the left, and an electrically stimulated tissue on the right undergoing 1 h of control, 3 h of electrical stimulation to the right, and 6 h of unstimulated relaxation.

Movie 2 (separate file). Phase-contrast time-lapse images of 0.5 mm-tall strips through the center of the two tissues which appear in Movie 1, providing a sharper video of the dynamics through the entire width of the tissues. Here, the strip of the control tissue appears on top, through its 10 h of unperturbed growth, and the strip of the electrically stimulated tissue appears on bottom, through the three phases of the experiment—1 h of unstimulated control time, 3 h of electrical stimulation 'rightward', and 6 h of relaxation time post-stimulation.

Movie 3 (separate file). Heatmaps of velocity parallel to (V_x) and orthogonal to (V_y) the direction of stimulation through our entire timelapse. The data here is garnered from PIV data (see Methods) for the same two tissues which appear in Movie 1. 10 h of a control tissue appear in the top two panels, and the electrically stimulated tissue appears in the bottom two panels, again through 1 h unstimulated, 3 h electrical stimulation to the right, and 6 h relaxation post-stimulation.

Movie 4 (separate file). Time-lapse of a sample portion of the top edge of a stimulated tissue, with sample TrackMate tracks superimposed to highlight the apparent recoil in this area of the tissue. The image itself was generated using our in-house Fluorescence Reconstruction Microscopy tool (see Methods).

Movie 5 (separate file). Time-lapse of E-cadherin:DsRed MDCK cells during our bioelectric assay with three different regions of cellular tissue. Most notably, we included an area of single cells and clusters (lower panel), in addition to the bulk (left panel) and edge (right panel) regions. Cells in all regions show vertical polarization, perpendicular to the field direction (horizontal), during stimulation. However, single cells and clusters still do not show electrotactic migration, confirming past literature. The epithelial cell clusters detach from one another during electrical stimulation, and only seem to form clusters again near the end of our 10 h assay.

Movie 6 (separate file). Strain wave propagation in both directions for control and stimulated tissues. In control tissues in the top two panels, strain waves are seen natively moving throughout the tissue; in stimulated tissues however, a particularly stark wave traveled inward from each edge, beginning at the end of stimulation—on the right (leading) tissue edge in x-strains and the top and bottom tissue edges in y-strains. Additionally, in the bulk of the y-strain panel, careful inspection shows strain waves during stimulation moving rightward, even as they propagate vertically, as discussed in the main text with Figs. 5D-E.
